# Supplementary material for: Converting waste PET plastics into automobile fuels and antifreeze components
Source: Nat Commun. 2022 Jun 10;13:3343. doi: 10.1038/s41467-022-31078-w (PMC9187643; doi:10.1038/s41467-022-31078-w)
Supplement: Supplementary file 1 — Supplementary Information [file 41467_2022_31078_MOESM1_ESM.pdf]

Supplementary Information

Converting waste PET plastics into automobile fuels and antifreeze components

Zhiwen Gao[12], Bing Ma[12*], Shuang Chen[12], Jingqing Tian[12], Chen Zhao[12*]

[1] Shanghai Key Laboratory of Green Chemistry and Chemical Processes, School of Chemistry and Molecular Engineering, East China Normal University, Shanghai 200062, China.

[2] Institute of Eco-chongming, Shanghai 202162, China.

Email: bma@chem.ecnu.edu.cn;

czhao@chem.ecnu.edu.cn

Inventory of Supplementary Information

Supplementary Methods 3

Chemicals 3

Catalyst preparations 3

Catalyst characterizations 4

Supplementary Figures 7

Supplementary Tables 25

Supplementary Notes 38

Supplementary References 39

Supplementary Methods

Chemicals

The following chemicals were received from a commercial supplier and directly used without any pretreatment: nano silicon dioxide (SiO_2, ⩾99.9%, Sinopharm Chemical Reagent Co., Ltd.), ammonium chloride (NH_4Cl, ⩾99.8%, Sinopharm Chemical Reagent Co., Ltd.), sodium chloride (NaCl, ⩾99.8%, Sinopharm Chemical Reagent Co., Ltd.), polyethylene terephthalate (PET, Sinopharm Chemical Reagent Co., Ltd.), copper nitrate ($Cu(NO_3)_2 \cdot 3H_2O$, 99.0～102.0%, Sinopharm Chemical Reagent Co., Ltd.), ammonia ($NH_3 \cdot H_2O$, AR, Sigma-Aldrich), lithium chloride ($LiCl \cdot H_2O$, ⩾97.0%, Sinopharm Chemical Reagent Co., Ltd.), chlorinated potassium (KCl, 99.8%, Aladdin), rubidium chloride (RbCl, 99.95%, Aladdin), cesium chloride (CsCl, 99.99%, Aladdin), cobalt nitrate ($Co(NO_3)_2 \cdot 3H_2O$, 99%, Aladdin), nickel nitrate ($Ni(NO_3)_2 \cdot 3H_2O$, ⩾98.0%, Sinopharm Chemical Reagent Co., Ltd.), iron nitrate ($Fe(NO_3)_3 \cdot 9H_2O$, ⩾98.5%, Sinopharm Chemical Reagent Co., Ltd.), dimethyl terephthalate ($C_{10}H_{10}O_4$, 99%, Sigma-Aldrich), methyl 4-(hydroxymethyl)benzoate ($C_9H_{10}O_3$, 98%, Accela), methyl *p*-toluate ($C_9H_{10}O_2$, 99%, Inno-CHEM), 4-methylbenzyl alcohol ($C_8H_{10}O$, ⩾99%, Inno-CHEM), titanium dioxide (TiO_2, ⩾98.0%, Sinopharm Chemical Reagent Co., Ltd.), zirconium dioxide (ZrO_2, ⩾99.0%, Sinopharm Chemical Reagent Co., Ltd.), and cerium dioxide (CeO_2, ⩾99.9%, Sinopharm Chemical Reagent Co., Ltd.).

Catalyst preparations

Synthesis of Cu/SiO_2 using the hydrothermal method. $Cu(NO_3)_2 \cdot 3H_2O$ was dissolved in deionized water, and a solution of NH_4Cl, $NH_3 \cdot H_2O$, and SiO_2 was added to the fully dissolved solution, stirred at room temperature for 0.5 h, and ultrasonically treated for 0.5 h. The mixture was then transferred to a polytetrafluoroethylene autoclave and kept at 120°C in a homogeneous reactor for 3 h. After cooling to room temperature, the solid in the autoclave was filtered and washed with deionized water until pH = 7 was reached. The conductivity of the liquid after last filtration was

4.2±0.3 μS cm^{-1}. This part has been supplemented into the supporting information part. The obtained precursor was dried overnight, calcined at 450°C in an air atmosphere for 4 h (air flow rate: 150 mL·min^{-1}, heating rate: 2°C·min^{-1}), and then reduced at 450°C in a hydrogen atmosphere for 4 h (hydrogen flow rate: 150 mL·min^{-1}, heating rate: 2°C·min^{-1}).

Catalyst characterizations

Powder X-ray diffraction (XRD): The crystal structure of the sample was characterized using a Japanese Rigaku Ultima IV X-ray diffractometer. The scanning parameters were: Cu K_{α} X-ray source, λ = 1.5406 Å, tube voltage of 35 kV, tube current of 25 mA, scanning range of 5–80°, scanning speed of 5°·min^{-1}, and scanning step of 0.02°.

Nitrogen physical adsorption (N_2-isotherm): a Quantachrome Autosorb-3B adsorption instrument was used for nitrogen physical adsorption testing. Before the measurement, the sample was activated under vacuum at 300°C for 3 h, and then nitrogen was adsorbed at liquid nitrogen temperature (77 K). The Brunauer–Emmet–Teller method was used to calculate the specific surface area, and the pore size distribution was analyzed by the Barrett–Jovner–Halenda method.

Transmission electron microscopy (TEM): Concerning on the TEM conditions, the samples prepared by grinding and subsequent dispersing the powder in ethanol and applying a drop of very dilute suspension on carbon-coated grids. The samples morphology and particle sizes were measured by a transmission electron microscopy (TEM) at 300 kV using a FEI Tecnai G2 F30 microscope.

The skeleton structures of the Fourier-Transform infrared spectroscopy (FTIR): catalyst samples were measured using a Nicolet Nexus 670 Fourier-transform infrared spectrometer. The test resolution was 4 cm^{-1}, the test wavenumber range was

400–4000 cm^{-1}, and the number of scans was 64. Before the measurement started, background collection (empty sample cell) was required.

FTIR measurement in vacuum: catalyst samples were measured using a Nicolet IS50 Fourier-transform infrared spectrometer. 8 mg sample powder was sufficiently ground with 20 mg KBr and pressed into a self-supporting disc (about 2.5 cm^2 area). Heated in situ up to 200 °C under vacuum and kept for 2 h, then cooled down to room temperature °C. Before the DRIFT measurements, the background spectrum was recorded under vacuum at the same resolution and number of scans.

Infrared spectroscopy (CO-FTIR): The metal species on the surface of the catalyst sample were measured by a Bruker Tensor 27 Fourier-transform infrared spectrometer (equipped with a Harrick diffuse reflectance accessory), and CO was used as the probe molecule for diffuse reflectance infrared spectroscopy. Diffuse reflectance infrared Fourier-transform infrared spectroscopy (CO-DRIFTS) was used for the measurements. Before any measurement, the sample was activated by argon (Ar) at 450°C for 30 min. After cooling to room temperature, a background scan was performed. The test wavenumber range was 600–4000 cm^{-1}, the number of scans was 64, and CO gas was used for maintenance. After 30 min, argon gas was passed in for purging, and the scan curve was recorded at the same time.

In-situ FTIR measurements: Performed by Bruker Tensor 27 Fourier-transform infrared spectrometer with a Harrick cell and an MCT detector. Before the experiment, the sample was pre-treated in the in-situ cell in an H_2 atmosphere at 450 °C for 2h, and then switched to an Ar atmosphere for 30 minutes to eliminate surface adsorbed substances. Then the background scan of the sample was performed. DMT and intermediates were dissolved in methanol and bubbled into the *in-situ* cell under Ar atmosphere to react for 6 h and collect the sample spectra every 1 h.

Thermogravimetric analysis (TGA): the weight loss curves of the catalyst samples were obtained on a NETZSCH STA449F3 (simultaneous TGA). The test temperature range was set to 30–1200°C, and the heating rate was 10°C·min^{-1}. High-purity air was used as the carrier gas, and the air flow rate was 50 mL·min^{-1}.

Hydrogen-temperature-programmed reduction (H_2-TPR): The calcined sample was characterized by temperature-programmed reduction on an advanced TP-5080 adsorption instrument (equipped with a thermal conductivity detector (TCD) detector). The gas product was a mixture of 5% H_2/He. The heating rate was 5°C·min^{-1}.

X-ray photoelectron spectroscopy (XPS): The valence state of the metal Cu on the surface of the catalyst was measured with a Thermo Scientific K-Alpha X-ray photoelectron spectrometer. Al Kα was selected as the X-ray emission source (hν = 1486.6 eV). The binding energy (BE) was corrected for surface charging by taking the C 1s peak of contaminant carbon as a reference at 284.58eV. Wagner – database of experimental sensitivity factors be used, based on F1s = 1.

Inductively coupled plasma atomic emission spectroscopy (ICP-AES): The content of each element in the catalyst sample was determined by a PerkinElmer Optima 8300 inductively coupled plasma atomic emission spectrometer. The test process was as follows, Firstly, the catalyst sample was dissolved in hydrofluoric acid to ensure that it was completely dissolved and in a clear state. Finally, the solution was diluted to a suitable test range. The five standard solutions were prepared to construct the external standard curve. The content of elements in the samples was determined by external standard curve.

Supplementary Figures

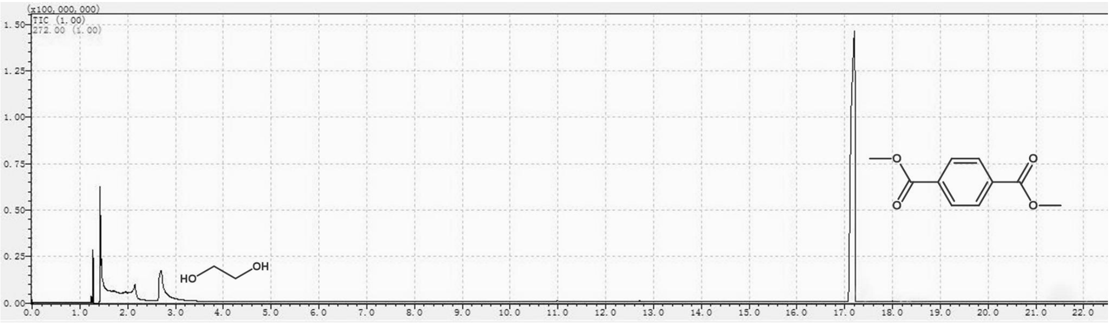

Supplementary Fig. 1 Gas chromatography (GC) spectrum of the liquid products after the degradation of polyethylene terephthalate (PET). Reaction conditions: PET (0.12 g), CH_3OH (30 mL); 210 °C with stirring at 600 rpm, 0.5 h.

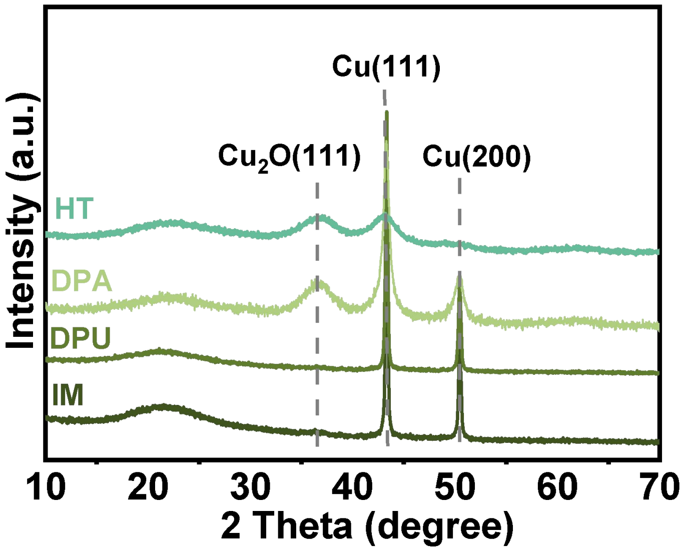

Supplementary Fig. 2 XRD patterns of Cu/SiO_2 (reduced) prepared by hydrothermal method (HT), deposition–precipitation with ammonia (DPA), deposition–precipitation with urea (DPU), and an impregnation method (IM).

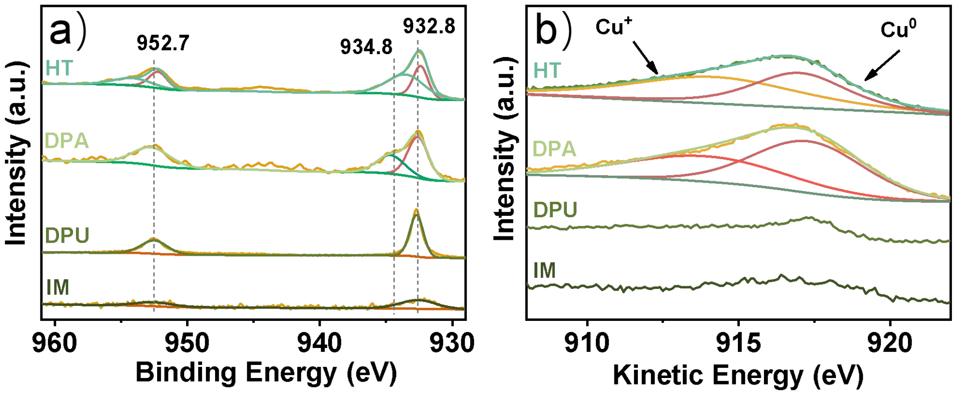

Supplementary Fig. 3 (a) XPS and (b) Cu LMM XAES profiles of Cu/SiO_2 (reduced) prepared by hydrothermal method (HT), deposition–precipitation with ammonia (DPA), deposition–precipitation with urea (DPU), and impregnation method (IM).

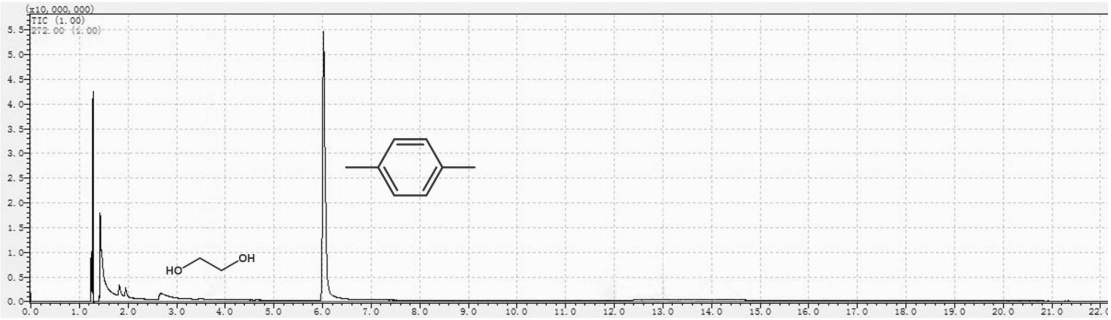

Supplementary Fig. 4 GC spectrum of the liquid products after conversion of PET. Reaction conditions: PET (0.12 g), CuNa/SiO_2 (0.1 g), CH_3OH (30 mL); 210 °C with stirring at 600 rpm, 6 h.

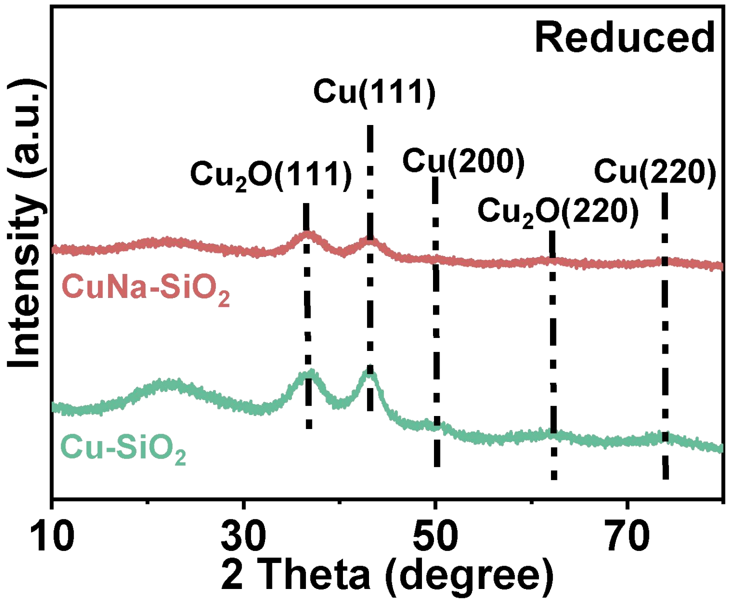

Supplementary Fig. 5 XRD patterns of reduced Cu/SiO_2 and CuNa/SiO_2.

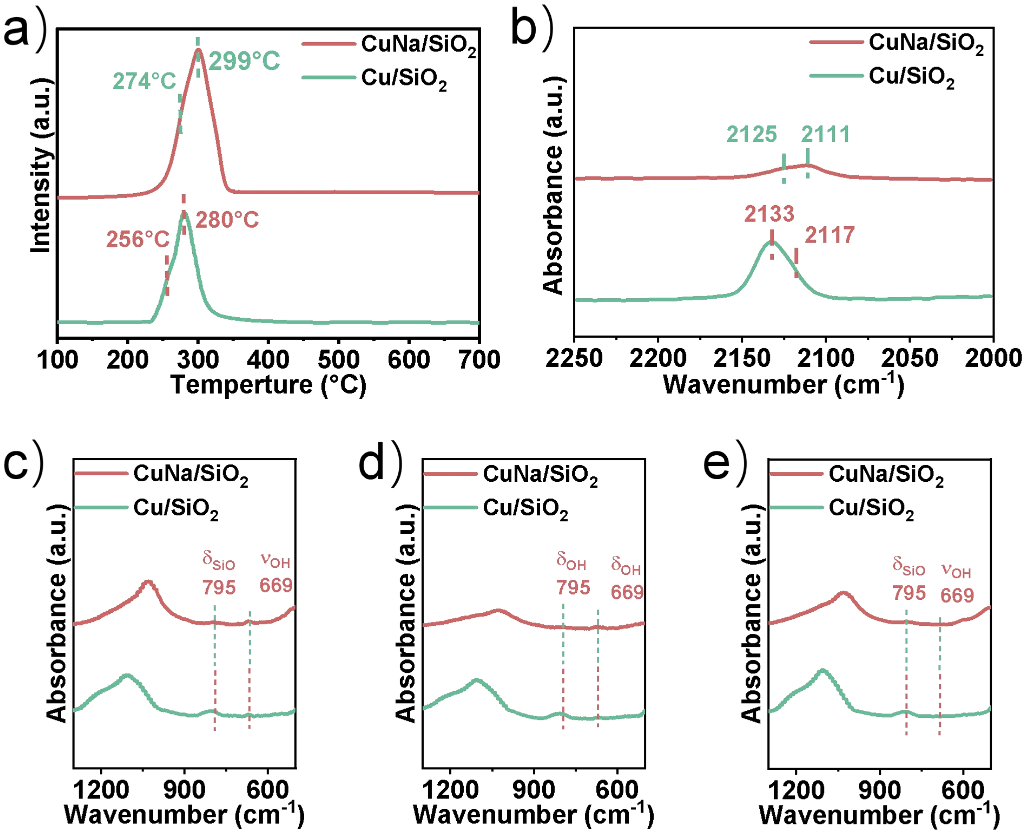

Supplementary Fig. 6 (a) Hydrogen temperature programmed reduction (H_2-TPR) profiles of Cu/SiO_2 and CuNa/SiO_2. (b) Fourier-transform infrared spectroscopy (FTIR) spectra for CO adsorption on Cu/SiO_2 and CuNa/SiO_2. FTIR spectra of (c) dried, (d) calcined, and **e** reduced Cu/SiO_2 and CuNa/SiO_2.

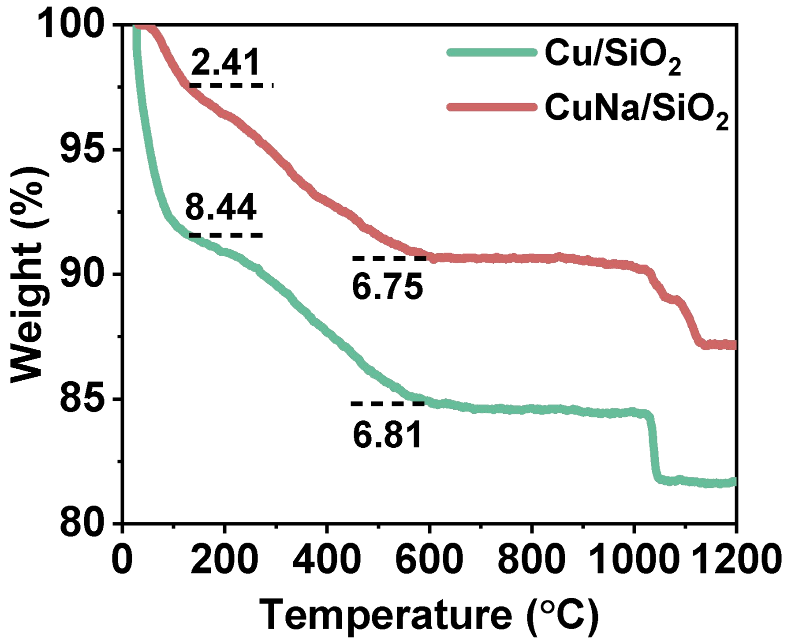

Supplementary Fig. 7 Thermogravimetric analysis (TGA) patterns of dried Cu/SiO_2 and CuNa/SiO_2 samples.

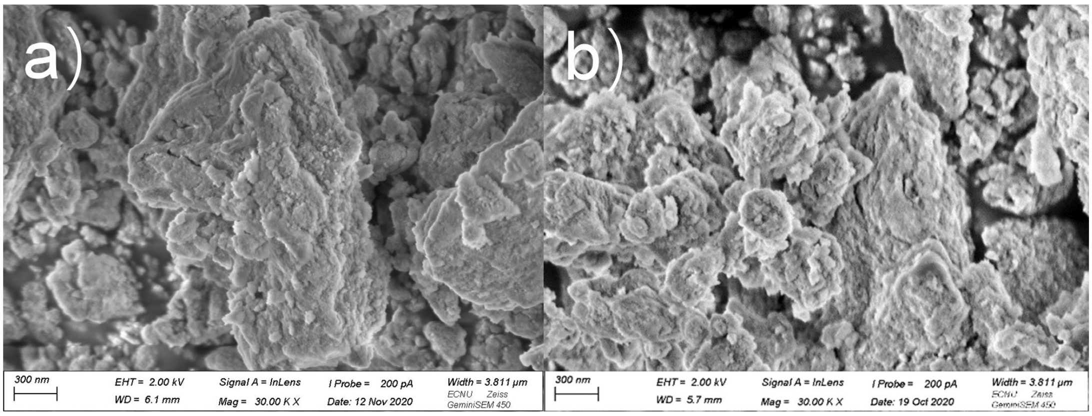

Supplementary Fig. 8 Scanning electron microscopy (SEM) images of: (a) Cu/SiO_2 (reduced) and (b) $CuNa/SiO_2$ (reduced).

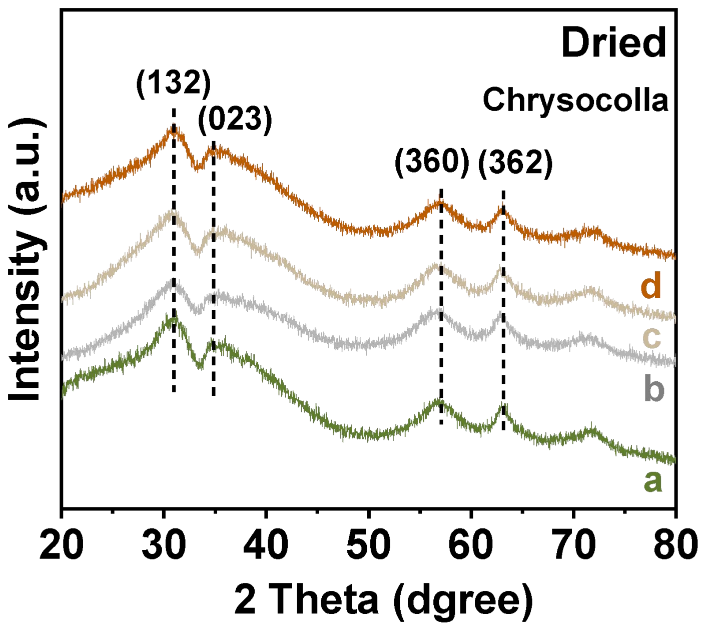

Supplementary Fig. 9 X-ray diffraction (XRD) patterns of dried samples: **a** Cu-2.5Na/SiO_2, **b** Cu-5Na/SiO_2, **c** Cu-10Na/SiO_2, and **d** Cu-15Na/SiO_2.

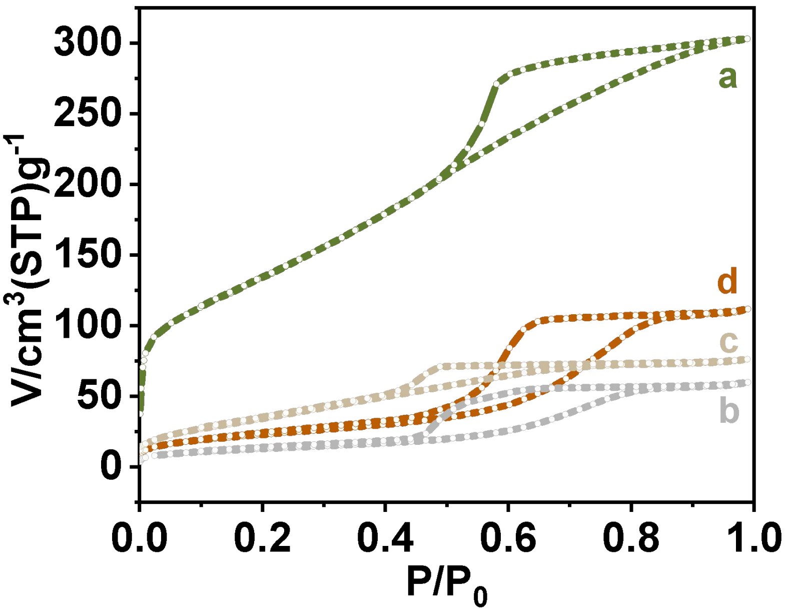

Supplementary Fig. 10 N_2 adsorption–desorption of dried samples: **a** Cu-2.5Na/SiO_2, **b** Cu-5Na/SiO_2, **c** Cu-10Na/SiO_2, and **d** Cu-15Na/SiO_2.

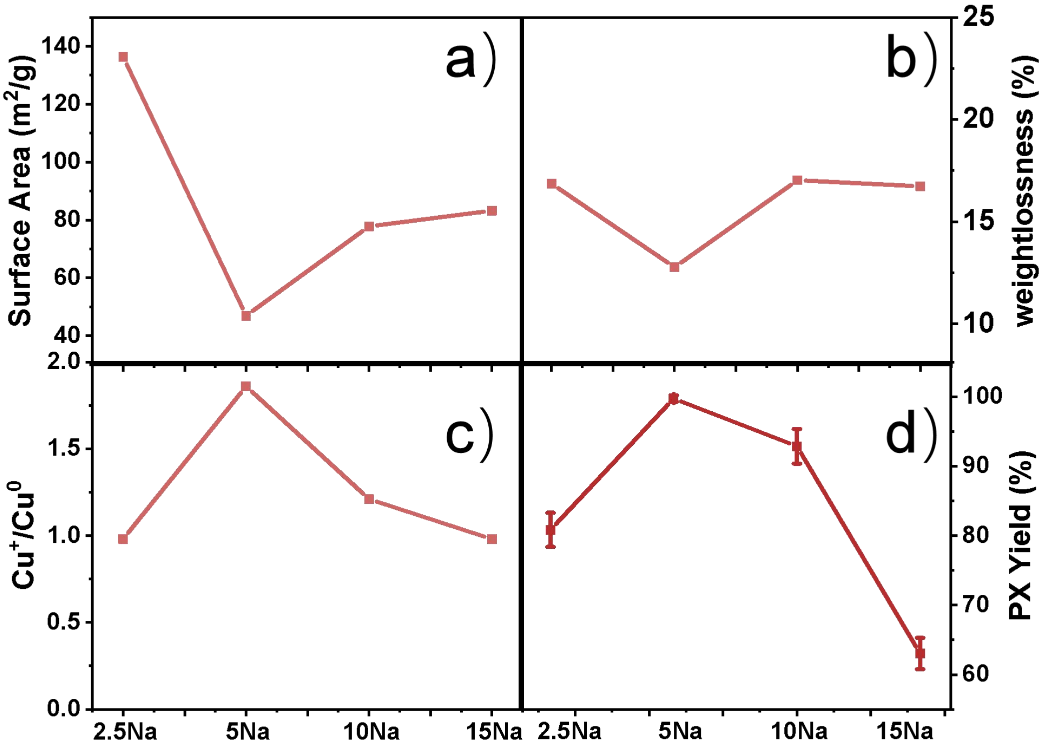

Supplementary Fig. 11 Effect of different contents of introduced NaCl on: (a) surface area of the copper silicate precursor. (b) weight loss of the copper silicate precursor. (c) Cu^{+}/Cu^{0} of the reduced sample and (d) *p*-xylene (PX) yield. Reaction conditions: PET, 0.12 g; CuNa/SiO_2 catalyst, 0.1 g; methanol, 30 mL; 210°C; 6 h. Data are presented as mean ± s.d. of three independent experiments.

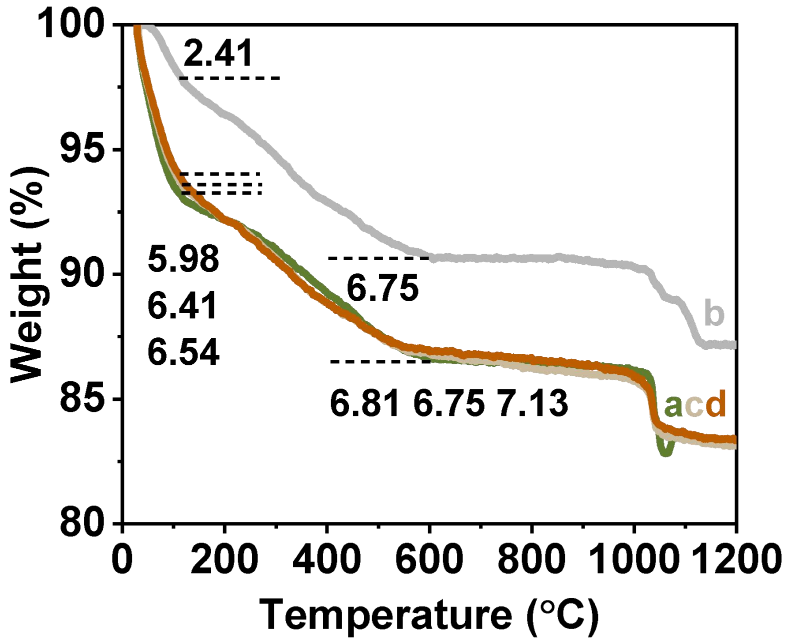

Supplementary Fig. 12 TGA patterns of dried samples: **a** Cu-2.5Na/SiO_2, **b** Cu-5Na/SiO_2, **c** Cu-10Na/SiO_2, and **d** Cu-15Na/SiO_2.

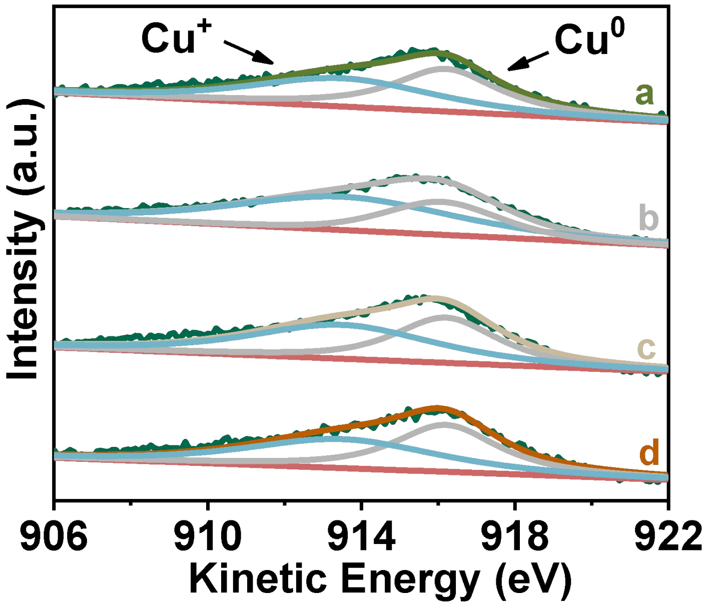

Supplementary Fig. 13 Cu LMM X-ray induced Auger electron spectroscopy (XAES) spectra of reduced samples: **a** Cu-2.5Na/SiO_2, **b** Cu-5Na/SiO_2, **c** Cu-10Na/SiO_2, and **d** Cu-15Na/SiO_2.

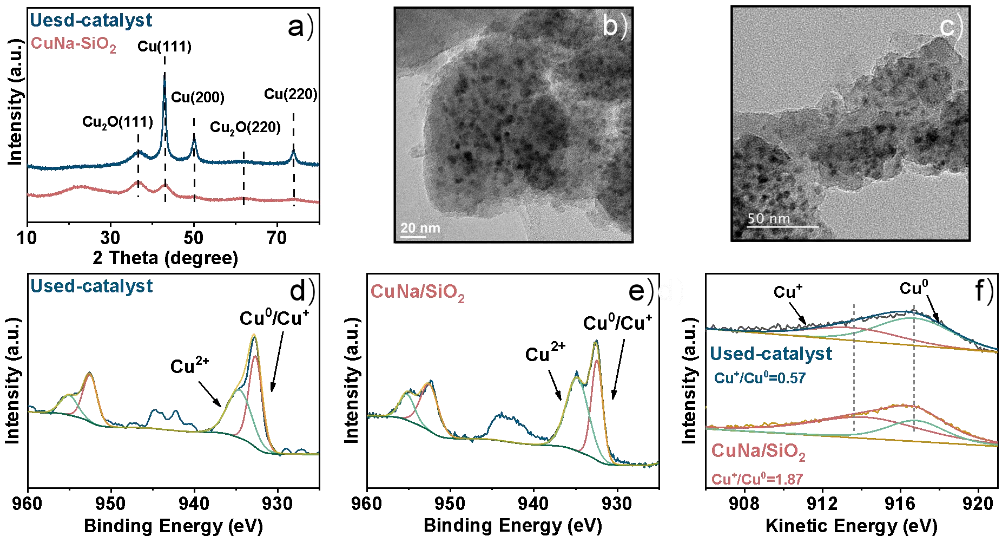

Supplementary Fig. 14 (a) XRD patterns of used CuNa/SiO_2 catalyst and CuNa/SiO_2 (reduced); TEM image of (b) used CuNa/SiO_2 catalyst and (c) CuNa/SiO_2 (reduced); XPS Cu 2*p* spectra of (d) used CuNa/SiO_2 catalyst and (e) CuNa/SiO_2 (reduced); (f) Cu LMM XAES spectra of used CuNa/SiO_2 catalyst and CuNa/SiO_2 (reduced).

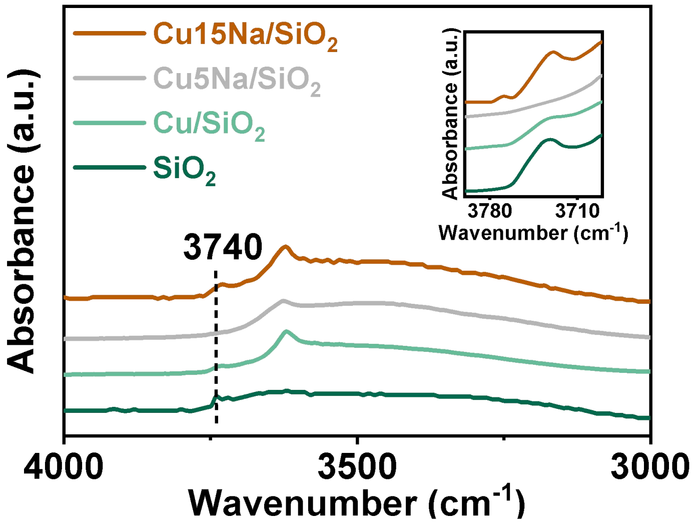

Supplementary Fig. 15 IR spectra in a vacuum of SiO_2, Cu/SiO_2, $Cu5Na/SiO_2$, and $Cu15Na/SiO_2$.

In order to roughly quantify the interface areas of the formed copper silicate with SiO_2, we supplemented the measurement of IR spectroscopy in vacuum and determined the remaining silanols groups on SiO_2. The peak at 3740 cm^{-1} in the SiO_2 sample is attributed to Si-OH groups (Supplementary Fig. 15)[1, 2]. With the traditional hydrothermal method, Cu^{2+} in the solution combined with the silanol on the SiO_2 surface to form copper silicate, which accelerated the layered copper silicate nucleation and growth significantly. Thus only a small amount of Si-OH can still be detected. Upon addition of 5 NaCl, a large amount of Na^+ occupied the silanol on the surface of the SiO_2, Cu^{2+} in the solution could only be combined with the remaining silanol on the SiO_2 surface to form scattered and isolated copper silicate particles (Figures 2g and 2i), which was attached to the surface of the carrier. Thus, the formed granular copper silicate showed a large interface area with SiO_2, since no remaining Si-OH on $CuNa/SiO_2$ was detected via IR measurement in vacuum (Supplementary Fig. 15). When 15 Na^+ was introduced, Na^+ occupied almost all the Si-OH on the surface, and Cu^{2+} can only combined with SiO_3^{2-} in the solution to form granular copper silicate and then deposited on the SiO_2. After washed with deionized water, all Si-OH groups are exposed, the peak at 3740 cm^{-1} is basically reserved. Therefore, this type of copper silicate showed better crystallinity (Supplementary Table 7) and small interface areas with SiO_2 (Supplementary Fig. 15).

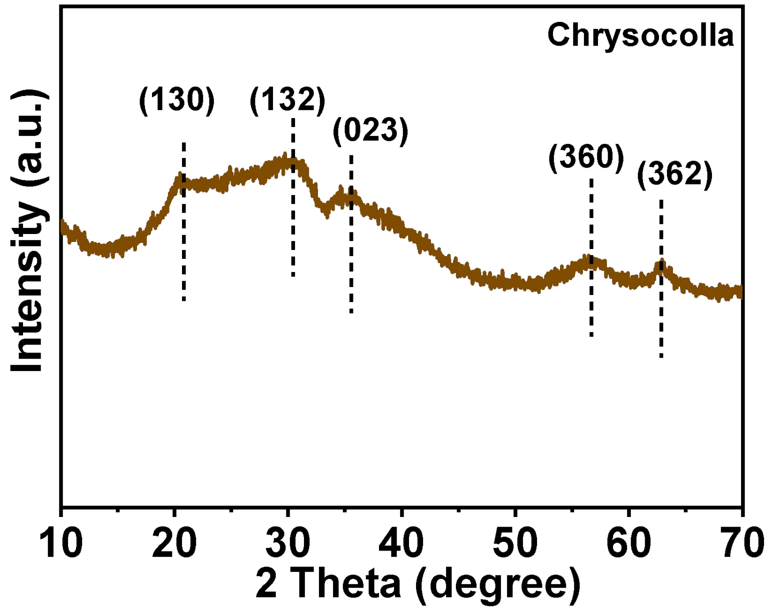

Supplementary Fig. 16 XRD patterns of Cu/SiO_2-HT-Na-IM (dried).

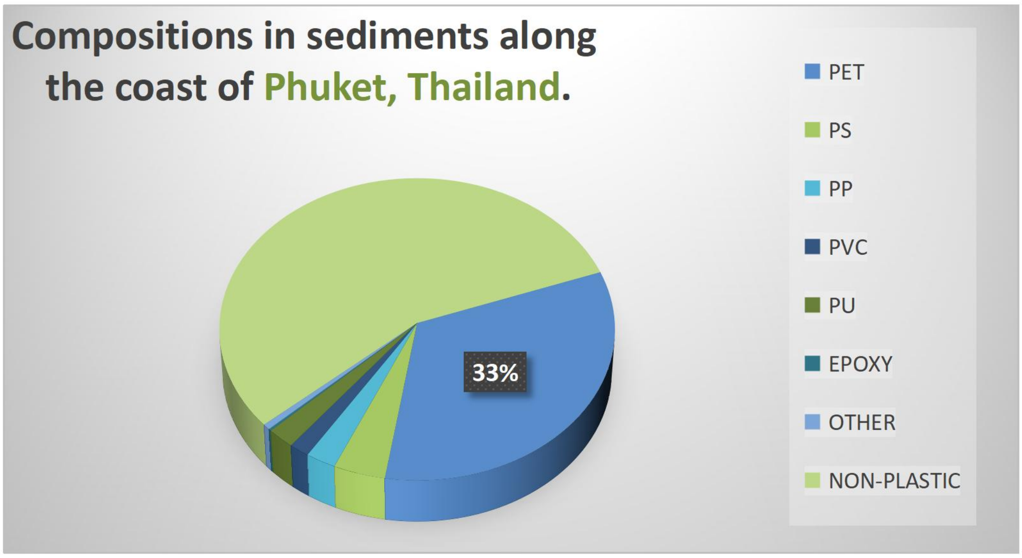

Supplementary Fig. 17 Compositions in sediments along the coast of Phuket island.

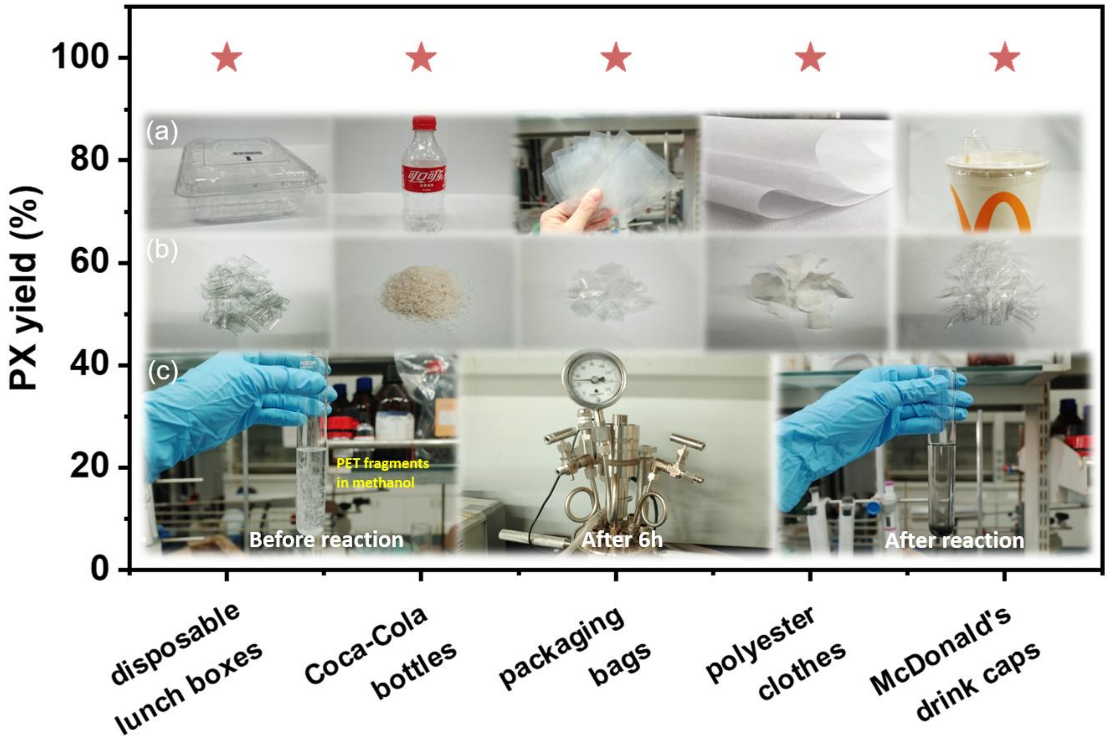

Supplementary Fig. 18 (a) Disposable lunch boxes, Coca-Cola bottles, packaging bags, polyester clothes and McDonald's drink caps. (b) Fragments of the plastics after processing. (c) The snapshots of the reaction system.

Supplementary Tables

Supplementary Table 1. PET alcoholysis at different temperatures in methanol.

Temperature (°C)	PET Conv. (%)	DMT yield (%)
210	100	100
200	100	100
190	78.6	78.6
180	63.3	63.3
170	19.8	19.8

Reaction conditions: 0.12 g PET, 30 mL methanol, 30 min.

Supplementary Table 2. PET conversion over $CuNa/SiO_2$ catalyst at different temperatures in methanol.

T (°C)	PET Conv. (%)	DMT yield (%)	PX yield (%)	By-product yield (%)	
				Methyl 4-methylbenzoate	4-methylbenzyl alcohol
210	100	0	100	0	0
200	100	0	93.6	6.1	0.3
190	100	0	53.8	32.4	13.8
180	100	99.4	0	0.6	0
170	63.9	63.9	0	0	0

Reaction conditions: 0.12 g PBT, 0.1 g $CuNa/SiO_2$, 30 mL methanol, 6 h.

Supplementary Table 3. PET conversion in different solvents of methanol, ethanol and isopropanol.

Substrate	Catalyst	Solvent	Time (h)	PET Conv. (%)	Monomer yield (%)	PX yield (%)	Incremental pressure at RT (MPa)
PET	-	Methanol	0.5	100	100	0	0
-	CuNa/SiO_2	Methanol	6	-	-	-	3.7
PET	CuNa/SiO_2	Methanol	6	100	100	100	3.4
PET	-	Ethanol	0.5	80.3	80.3	0	0
-	CuNa/SiO_2	Ethanol	6	-	-	-	0.7
PET	CuNa/SiO_2	Ethanol	6	97.3	97.3	0	0.7
PET	-	Isopropanol	0.5	73.5	73.5	0	0
-	CuNa/SiO_2	Isopropanol	6	-	-	-	0.8
PET	CuNa/SiO_2	Isopropanol	6	87.1	87.1	0	0.8

Reaction conditions: 0.12 g PET, 0.1 g CuNa/SiO_2, 30 mL solvent, 210 °C.

Supplementary Table 4. PBT conversion in methanol over CuNa/SiO_2 at different temperatures.

T (°C)	PBT Conv. (%)	DMT yield (%)	PX yield (%)	By-product yield (%)		Incremental pressure at RT (MPa)	Gas composition (%)			
				Methyl 4-methylbenzoate	4-Methylbenzyl alcohol		H_2	CO	CO_2	CH_4
210	100	0	100	0	0	2.8	60	36	-	4
200	100	0	95.7	2.2	2.1	2.2	62	35	-	5
190	100	0	60.6	23.6	15.8	1.5	60	36	-	4
180	100	98.6	0	1.4	0	0.5	55	39	1	5
170	72.4	72.4	0	0	0	0	-	-	-	-

Reaction conditions: 0.12 g PBT, 0.1 g CuNa/SiO_2, 30 mL methanol, 6 h.

Supplementary Table 5. Mass coverage (wt%) on the catalyst surfaces resulting from XPS (Cu2*p*) peak and Cu LMM XAES peak deconvolution.

Catalyst	XPS			XAES		
	T(Cu) [a]	Cu^{2+} [a]	$\frac{Cu^{2+}}{T(Cu)}$ [a]	Cu^{+} [b]	Cu^{0} [b]	$\frac{Cu^{+}}{Cu^{+}+Cu^{0}}$ [b]
Cu/SiO_2	4.68	1.73	0.37	1.66	1.29	0.56
$CuNa/SiO_2$	5.92	2.62	0.44	2.15	1.15	0.65

Note: [a] T(Cu) is the total surface content of Cu, obtained from XPS characterization.
[b] Intensity ratio between Cu^{+} and (Cu^{+}+ Cu^{0}) by deconvolution of Cu LMM XAES spectra.

Supplementary Table 6. Inductively coupled plasma (ICP) results of Cu/SiO_2 (dried) and $CuNa/SiO_2$ (dried).

Catalyst	Cu loading (wt.%)	Na Loading (wt.%)	PX yield (%)	Incremental pressure at RT (MPa)
Cu/SiO_2	65.9	0	73	2.9
$CuNa/SiO_2$	66.5	2.4	100	3.4

Reaction conditions: PET, 0.12 g; catalyst, 0.1 g; methanol, 30 mL; 210 °C; 6 h. PX: *p*-xylene; RT: room temperature.

Supplementary Table 7. The crystallinity of different Cu/SiO_2 catalysts.

Catalyst	Crystallinity (%)	R (%)
Cu/SiO_2	45.1	1.51
$Cu2.5Na/SiO_2$	45.0	1.56
$Cu5Na/SiO_2$	23.6	1.17
$Cu10Na/SiO_2$	27.0	1.40
$Cu15Na/SiO_2$	27.1	1.31

Supplementary Table 8. Mass coverage (wt%) on the catalyst surfaces resulting from XPS (Cu2*p*) peak and Cu LMM XAES peak deconvolution.

Catalyst	XPS		XAES		
	T(Cu) [a]	Cu^{2+} [a]	Cu^{+} [b]	Cu^{0} [b]	$\frac{Cu^{+}}{Cu^{+}+Cu^{0}}$ [b]
Cu2.5Na/SiO_2	4.38	2.46	0.95	0.97	0.49
Cu5Na/SiO_2	5.92	2.62	2.15	1.15	0.65
Cu10Na/SiO_2	4.61	2.31	1.26	1.04	0.55
Cu15Na/SiO_2	4.38	1.59	1.38	1.41	0.49

Note: [a] T(Cu) is the total surface content of Cu, obtained from XPS characterization.
[b] Intensity ratio between Cu^{+} and (Cu^{+}+ Cu^{0}) by deconvolution of Cu LMM XAES spectra.

Supplementary Table 9. Recycling tests of the conversion of PET over $CuNa/SiO_2$.

Run times	1	2	3	4
PX yield (%)	100	96.4	52.7	0

Reaction conditions: PET, 0.12 g; catalyst, 0.1 g; methanol, 30 mL; 210 °C; 6 h.

Supplementary Table 10. Inductively coupled plasma (ICP) results of $CuNa/SiO_2$-HT (dried) and Cu/SiO_2-HT-Na-IM (dried).

Catalyst	Cu loading (wt.%)	Na Loading (wt.%)	PX yield (%)	Incremental pressure at RT (MPa)
$CuNa/SiO_2$-HT	66.5	2.4	100	3.4
Cu/SiO_2-HT-Na-IM	67.2	2.4	65.8	2.7

Reaction conditions: PET, 0.12 g; catalyst, 0.1 g; methanol, 30 mL; 210 °C; 6 h. PX: *p*-xylene; RT: room temperature.

Supplementary Table 11. Summary of main results in literature and parallel works for PET conversion.

Catalyst	Noble metal	T (°C)	Reaction time (min)	Yield arene (%)	Solvent/PET mass ratio	Catalyst/PET mass ratio	Energy economy (ε) (°C^{-1}*min^{-1})	Environmental factor (a.u)	Environmental energy impact (ξ) (°C*min)	Ref.
Ru/Nb_2O_5	Yes	200	720	87.1	100.0	1.00	6.050E-6	23.74	3923967	3
Ru/Nb_2O_5	Yes	220	720	90.4	75.0	1.00	5.710E-6	19.70	3450088	4
$CuNa/SiO_2$	No	210	360	100	197.5	0.83	1.323E-5	37.19	2811035	This work
$CuNa/SiO_2$	No	210	360	100	98.75	0.83	1.323E-5	19.40	1466364	This work
$CuNa/SiO_2$	No	210	360	100	65.83	0.83	1.323E-5	13.41	1013605	This work

We tried to use the environmental factor and environmental energy impact in Thielemans et al.'s work to evaluate the efficiency of several parallel works[5]. Firstly, energy economy coefficient (ε) is proposed to enable objective comparison on the influence of para meters such as temperature, catalyst type, or proportion of starting materials, where t is the reaction time (in minutes), T is the reaction temperature in degrees celsius, and Y is the yield of the main monomer in mass fraction (which contains the aromatic moiety) in eqn (1). Thielemans et al. improved the environmental factor (E_{factor}) in eqn (4) which took the effect of materials input that results in waste generation into consideration. The environmental energy impact (ξ) results from the combination of the two factors above as presented in eqn (5). The best processes would tend to present high ε coefficient and low values of E_{factor} and ξ.

$$\varepsilon = \frac{Y}{T \times t} \quad (1)$$

$$E_{factor} = \frac{\left[0.1 \times \left(\frac{solvent}{PET} ratio\right) + \left(\frac{cat}{PET} ratio\right) + \left(other \frac{subst}{PET} ratio\right)\right] \times m_{PET}}{m_{Product}} \quad (2)$$

$$m_{Product} = yield_{Product} \times \frac{MM_{Product}}{MM_{PET\ MERE}} \times m_{PET} \quad (3)$$

Replacing (3) in (2)

$$E_{factor} = \frac{\left[0.1 \times \left(\frac{solvent}{PET} ratio\right) + \left(\frac{cat}{PET} ratio\right) + \left(other \frac{subst}{PET} ratio\right)\right] \times m_{PET}}{yield_{Product} \times \frac{MM_{Product}}{MM_{PET\ MERE}} \times m_{PET}} \quad (4)$$

$$\xi = \frac{E_{factor}}{\varepsilon} \quad (5)$$

Supplementary Table 12. Boiling points of different substances.

Substance	Boiling point (°C)
Methanol	64.7
p-Xylene	138.5
Ethylene Glycol	197.4

Supplementary Notes

Reactions involved in the PET conversion process:

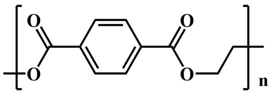

$$+ 6n\ H_2 \longrightarrow n\ PX + n\ EG + 2n\ H_2O$$

$$CH_3OH \rightarrow CO + 2H_2$$

$$CO + 3H_2 \rightarrow CH_4 + H_2O$$

Real methanol consumption (according to liquid phase)：

Experimental Method: 30 mL methanol was diluted 10 times in ethyl acetate with 0.1 mL tetrahydronaphthalene as the internal standard before the reaction and determined the response factor (*f*) by GC-MS using following formula.

$$f = \frac{A_S}{A_i} * \frac{V_i}{V_S}$$

The reacted solution was diluted 10 times with ethyl acetate, the residual amount of methanol is calculated by the following formula subsequently. According to the reduction of peak area, the residual methanol content was 97.6%.

$$V_{iu} = f * \frac{A_{iu}}{A_S} * V_S$$

$n_{consumption}(CH_3OH) = 2.4\% * n(CH_3OH_{original}) = 0.0203$ mol

Therefore, the consumption of methanol is 0.0203 mol.

Real methanol consumption (according to gaseous phase):

The composition of the gaseous phase after reaction:

H_2 (60%), CO (36%), CH_4 (4%),

298 K, 3.4 MPa, 35 mL volume of reactor

Carbon Balance:

$n_{consumption}(CH_3OH) = n(C_{gaseous\ phase})$

$= n(CO) + n(CH_4)$

$= 0.0192$mol

$n_{consume}(H_2) = 6n(PET)$

$=0.0038$mol

Hydrogen Balance:

$n_{production}(H_2) = n_{residual\ amout}(H_2) + n_{consume}(H_2)$

$= n(H_2) + 3n(CH_4) + 6n(PET)$

$= 0.0383\text{mol} \approx 2n_{consumption}(CH_3OH)$

Based on the above results, it can be concluded that the methanol consumption calculated from the product well matches the actual consumption of methanol and the generation of hydrogen.

Supplementary References

1. Tada H. Layer-by-layer construction of SiO_x film on oxide semiconductors. *Langmuir* **11**, 3281-3284 (1995).

2. Nakamura M, Kobayashi M, Kuzuya N, Komatsu T, Mochizuka T. Hydrophilic property of SiO_2/TiO_2 double layer films. *Thin Solid Films* **502**, 121-124 (2006).

3. Jing Y*, et al.* Towards the Circular Economy: Converting aromatic plastic waste back to arenes over a Ru/Nb_2O_5 catalyst. *Angewandte Chemie International Edition* **60**, 5527-5535 (2021).

4. Lu S, Jing Y, Feng B, Guo Y, Liu X, Wang Y. H_2-free plastic conversion: converting PET back to BTX by unlocking hidden hydrogen. *ChemSusChem* **14**, 4242-4250 (2021).

5. Barnard E, Rubio Arias JJ, Thielemans W. Chemolytic depolymerisation of PET: a review. *Green Chemistry* **23**, 3765-3789 (2021).
