# Supplementary material for: Converting waste PET plastics into automobile fuels and antifreeze components
Source: Nat Commun. 2022 Jun 10;13:3343. doi: 10.1038/s41467-022-31078-w (PMC9187643; doi:10.1038/s41467-022-31078-w)
Supplement: Supplementary file 3 — Description of Additional Supplementary Files [file 41467_2022_31078_MOESM3_ESM.pdf]

Description of Additional Supplementary Files

File Name: Supplementary Code 1
Description: Zip folder contains instructions and code for fitting reaction rate constants usingMATLAB.
